# A Model Nutrition Control System in Potato Tissue Culture and Its Influence on Plant Elemental Composition

**DOI:** 10.3390/plants11202718

**Published:** 2022-10-14

**Authors:** Chandiona Munthali, Rintaro Kinoshita, Kazumitsu Onishi, Aurelie Rakotondrafara, Kakeru Mikami, Masanori Koike, Masayuki Tani, Jiwan Palta, Daigo Aiuchi

**Affiliations:** 1 Department of Agro-Environmental Science, Obihiro University of Agriculture and Veterinary Medicine, Inada-cho, Obihiro 080-8555, Hokkaido, Japan; 2 Research Center for Global Agromedicine, Obihiro University of Agriculture and Veterinary Medicine, Inada-cho, Obihiro 080-8555, Hokkaido, Japan; 3 Department of Plant Pathology, University of Wisconsin-Madison, 1630 Linden Drive, Madison, WI 53706, USA; 4 Department of Horticulture, University of Wisconsin-Madison, 490 Moore Hall, 1575 Linden Drive, Madison, WI 53706, USA

**Keywords:** insufficient nutrient supply, excessive nutrient supply, tissue culture, *Solanum tuberosum* L., nutrient interactions

## Abstract

Low or excessive soil fertility is a major constraint to potato production. The influence of each individual nutrient element on potato plants under field studies remains ambiguous due to the influence of environmental variations. Creating an in vitro model plant with deficient or excessive nutrient content will provide a more controlled study and allow for a better understanding of how the concentration of one element can affect the uptake of other elements. Here we designed a tissue culture-based nutrition control system to systematically analyze the effects of essential nutrients on potato plants. Insufficient or excessive nitrogen (N), phosphorus (P), potassium (K), calcium (Ca), and magnesium (Mg) contents were created by modifying the Murashige and Skoog (MS) medium. Deficient to toxic plant nutrient statuses were successfully defined by the evaluation of dry biomass and morphological symptoms. The results showed that plant shoot growth, nutrient uptake and content, and nutrient interactions were all significantly impacted by the changes in the MS media nutrient concentrations. These tissue culture systems can be successfully used for further investigations of nutrient effects on potato production in response to biotic and abiotic stresses in vitro.

## 1. Introduction

Potato (*Solanum tuberosum* L.) is one of the world’s most important crops, providing major contributions to human nutrition, livestock feed, employment, and income [1]. Potato requires a variety of essential nutrients for growth and development, and intensive fertilizer applications remain the main viable option for improving yield and quality. Insufficient or excessive soil nutrient contents are known to influence plant nutrient levels and affect the sensitivity of potatoes to abiotic stresses such as heat and drought as well as biotic stresses such as diseases and insect pests [2]. For instance, Ca status in the potato root zone is known to regulate abiotic stresses including heat and frost [3]. Increases in leaf N content have been linked to an increased host suitability of the potato plants to leaf miners, whereas increases in P and K were detrimental to the insects [4]. Butler et al. [5] reported an overall positive response of insect pests to increased fertilization but noted that findings are still conflicting. The results of field studies can vary because nutrient availability is influenced by a variety of factors, including the soil’s complex chemical, physical, and biological properties, as well as their interactions with environmental factors such as temperature, radiation, and water supply. Therefore, research in a controlled environment may reveal clear interactions between plant nutrient status and biotic as well as abiotic stresses.

The tissue culture technique has been widely used for rapid large-scale multiplications of plants, including potatoes, in an aseptic and controlled environment. Murashige and Skoog (MS) medium, a plant growth medium developed based on the nutrient composition of tobacco leaves to propagate tobacco pith, has been widely used for tissue culture [6]. Manipulation of the nutrient composition in the MS medium could be used to produce potato plants with insufficient and excessive nutrition status of a particular element. Several studies have attempted to adjust MS medium nutrient concentrations for the particular purpose of increasing either plantlet growth or microtuber yield. Radouani and Lauer [7] simultaneously increased N, P, and K contents from the standard 60 mM, 1.25 mM, and 20 mM, respectively, in MS medium, which resulted in an elevated number of microtubers, microtuber weight, and stem and root weights. The purpose of this study was to increase microtuber yield, and the study did not measure the nutrient contents in the plants. Nguyen et al. [8] assessed potato growth under insufficient N levels of 0 to 7.5 and 60 mM and reported the highest shoot biomass at 7.5 mM. Upadhyaya et al. [9] assessed the growth of potato cultivar Desiree at Ca levels of 0 to 12 mM and reported the highest growth and tuber yield at 9 mM Ca. These studies found changes in the morphology of the plant by adjusting the nutrient concentrations. However, there are very few studies evaluating the nutrient contents that could alter visible plant growth or invisible plant functions.

Walworth and Muniz [10] provided a comprehensive review of several field-grown potato cultivars with insufficient and excessive whole potato leaf N, P, K, Ca, and Mg contents at flowering stage, and their relationship to yield. Geary et al. [11] established deficient, adequate, and excess N nutrient status in potato plants under hydroponic conditions by evaluating N content. No studies like this have been done for potato tissue culture, but the nutrient status has been successfully determined in tissue culture studies of other crops, including peach almonds, flowers, and legume trees [12,13,14]. The adoption of this approach may thus aid in growing potato plants with insufficient or excessive nutrient content in a tissue culture system.

Varying the rate of application of one element is known to affect the plant uptake of other elements. Major macronutrients have been found to interact with each other; i.e., N is known to interact with P, K, Ca, and Mg in many different plant species [15]. These interactions could either be antagonistic or synergistic either in the soil or within the plant in a complex manner. For example, both competitive and synergistic uptake of multiple nutrients from the soil can occur among the elements [16]. In addition, root growth can be enhanced by some nutrients, which can accelerate the uptake of other nutrients [17]. These intricate interactions occur in both soil and plants, affecting nutrient composition, yield, and quality of crops. Currently, there is a scarcity of information on nutrient interactions in potato plants in vitro, and the tissue culture system with varying nutrient concentrations in the MS media could be an effective way to study this, under a controlled environment.

In this study, a model tissue culture nutrient control system was designed based on the whole potato leaf’s nutrient contents in the field, and the effects of essential nutrient supply on potato growth and development were systematically analyzed. The objectives of the study were to (1) establish nutrient concentration levels in the media to produce potato plants with insufficient and excessive nutrient content and (2) evaluate the nutritional interactions driven by variations in nutrient element concentrations in the media. This data could be valuable in investigations into the effects of nutrients on biotic and abiotic stresses on potato production.

## 2. Materials and Methods

### 2.1. Plant Material and Growth Conditions

The potato, *Solanum tuberosum*, cv. Irish Cobbler, was used to generate plant materials in the entire experiment, utilizing a tissue culture approach. Disease-free stock plantlets were multiplied and maintained from aseptically excised 1 cm long leafless single-node sections in cup-closed 60 mL transparent glass tubes containing 10 mL of standard MS medium, which contained MS inorganic salts, Fe-EDTA, H vitamins, 30 g L^−1^ of sucrose, and 7 g L^−1^ of Bacto Agar [6]. The MS inorganic salts contained 0.83 mg L^−1^ of potassium iodide (KI), 6.2 mg L^−1^ of boric acid (H_3_BO_3_), 8.6 mg L^−1^ of zinc sulfate heptahydrate (ZnSO_4_·7H_2_O), 24.1 mg L^−1^ of manganese (II) sulfate pentahydrate (MnSO_4_·5H_2_O), 0.17 g L^−1^ of potassium dihydrogen phosphate (KH_2_PO_4_), 1.65 g L^−1^ of ammonium nitrate (NH_4_NO_3_), 1.9 g L^−1^ of potassium nitrate (KNO_3_), 0.44 g L^−1^ of calcium chloride dihydrate (CaCl_2_·2H_2_O), 0.37 g L^−1^ of magnesium sulfate heptahydrate (MgSO_4_·7H_2_O), 0.25 mg L^−1^ of disodium molybdate (VI) dehydrate (Na_2_MoO_4_·2H_2_O), 0.025 mg L^−1^ of copper (II) sulfate pentahydrate (CuSO_4_·5H_2_O), and 0.025 mg L^−1^ of cobalt (II) chloride hexahydrate (CoCl_2_·6H_2_O). The Fe-EDTA was composed of 46.60 mg L^−1^ of disodium ethylenediaminetetraacetate dihydrate (C_10_H_14_O_8_N_2_Na_2_·2H_2_O) and 34.75 mg L^−1^ of iron (II) sulfate heptahydrate (FeSO_4_·7H_2_O). The H vitamins were composed of 100 mg L^−1^ of myo-inositol, 2 mg L^−1^ of glycine, 0.5 mg L^−1^ of thiamine hydrochloride (vitamin B1 hydrochloride), 0.5 mg L^−1^ of pyridoxine hydrochloride (vitamin B6 hydrochloride), 5 mg L^−1^ of nicotinic acid, 0.05 mg L^−1^ of biotin, and 0.5 mg L^−1^ of folic acid. Subsequently, the multiplied disease-free plantlets were used to generate plantlets with insufficient and excessive N, P, K, Ca, and Mg contents, and five replicates were randomly selected for subsequent assessments. About 1 cm of leafless single-node sections were aseptically excised and inserted into cup-closed 60 mL transparent glass tubes containing 30 mL of MS media of different individual amounts of N, P, K, Ca, and Mg (Table 1). Then, the cup was opened when the shoots reached the mouth of the tube to allow the plantlets to develop 5 to 6 leaves outside the tube before further assessments. To prevent contamination from the outside and also moisture loss, sterilized glass beads (ca. 1 mm in diameter) and activated carbon were added to the media surface to a depth of about 0.7 mm and 0.5 mm, respectively. The approximate entire plant growth period was 5 to 6 weeks depending on the individual nutrient and supply amounts. All the plantlets were grown in a controlled room with the temperature maintained at 20 ± 2 °C with 16 h of light and 8 h of darkness throughout the entire experimental period. All the media had their pH adjusted to 5.6 and were autoclaved at 121 °C for 20 min before use.

### 2.2. Calculations of MS Media N, P, K, Ca, and Mg Concentrations

The N, P, K, Ca, and Mg concentrations in the MS media were manipulated to various concentrations using the insufficient and excessive N, P, K, Ca, and Mg contents provided by Walworth and Muniz [10] for the whole leaf of field-grown potatoes at flowering stage and formulas provided by Terrer and Tomas [14]. The excessive amount of each nutrient in the new media was calculated using Formula (1)

(1) $$Em=B\times \frac{\sum M}{\sum El}$$

where *Em* is the amount (mg L^−1^) of the excessive nutrient in the new media; *B* is the excessive content (%) of each nutrient in the potato whole leaf defined by [10]; *∑M* is the total sum of N, P, K, Ca, and Mg (mg L^−1^) in the standard MS medium; and ∑*El* is the total sum of the excessive content of N, P, K, Ca, and Mg in potato whole leaf. The deficient amounts (mg L^−1^) of each nutrient were calculated using Formula (2)

(2) $$Dm=Em\times \frac{\sum Dl}{\sum El}$$

where *Dm* is the deficient amount (mg L^−1^) of the nutrient in the new media; *Em* is the calculated excessive amount (mg L^−1^) of each nutrient in the new media (Formula (1)); ∑*Dl* is the total sum of deficient N, P, K, Ca, and Mg (mg L^−1^) in potato whole leaf defined by Walworth and Muniz [10]; and ∑*El* is the total sum of the excessive content of N, P, K, Ca, and Mg in potato whole leaf [10]. Further adjustments were made to the calculated nutrient concentrations when potato plantlets exhibited severe deficiency or toxicity symptoms to ensure asymptomatic plantlets can be produced. In the MS media, only the MS inorganic salts were changed according to the formulas above, while other nutrients, H vitamins, Fe-EDTA, sucrose, and Bacto Agar were kept the same as in the standard MS medium (Table 1).

### 2.3. Plant Growth Assessments

In order to understand the effects of the manipulated nutrient supply on potato growth in vitro, the fresh and dry weights of aerial biomass (stem plus leaves), hereafter referred to as fresh and dry shoot biomass, were measured on plantlets with 5 to 6 fully grown leaves outside the growth glass tubes. Five plantlets of uniform growth were randomly selected for each nutrient type and concentration for measurements of fresh and dry biomass weight using a digital scale. The fresh weights were measured immediately after sample collection, and the dry weights were determined after oven drying at 60 °C for 4 days. The plant’s morphological characteristics were visually observed to identify severe nutrient deficiency or toxicity symptoms. In this study, dry shoot biomass and morphological symptoms were used to define “deficient”, “insufficient”, “optimum”, “excessive”, and “toxic” levels. Deficient level was concluded when the visible symptoms of nutritional deficiency were observed. Insufficient level correlated with a significantly low dry shoot biomass. The optimum level was within the range of maximum biomass. Excessive level was concluded when, despite an increase in nutrient concentration, no additional increase in biomass was observed. Toxic level was when visible symptoms of nutrient toxicity were observed.

### 2.4. Plant Nutrient Composition Analyses

The nutrient composition was determined in the shoots of 5 individual plantlets as 5 replicates per each treatment after oven drying and fine grinding. Total nitrogen was measured using a dry combustion method by a CHN automated elemental analyzer (Vario EL III, Elementar Analysensyteme, Hanau, Germany). To determine shoot P, K, Ca, and Mg, approximately 0.01 g of fine ground sample was ashed using a muffle furnace followed by dissolution of the ash with 1 mol L^−1^ hydrochloric acid (HCl) according to Miller [18]. The digest was measured using an inductively coupled plasma atomic emission spectrometer (ICPE-9820, Shimadzu Corporation, Kyoto, Japan). Five replicates (potato plantlets) were analyzed for each treatment. These analytical machines were part of the Obihiro University of Agriculture and Veterinary Medicine Common Equipment. All measurements were calculated on a dry matter basis. The nutrient uptake was calculated by multiplying the shoot nutrient content by the dry shoot biomass.

### 2.5. Statistical Analyses

All statistical analyses on plant growth parameters and nutrient composition were performed using JASP statistical software version 0.15 [19]. Data on plant growth and nutrient composition were subjected to one-way analysis of variance followed by Tukey’s honestly significant difference to separate the means at *p* < 0.05. To check whether changing one nutrient causes a change in the uptake of other nutrients, the Bayesian Pearson’s correlations were tested on measured shoot nutrient uptake. The Bayes factor (BF_10_) values were used to categorize the strength of evidence as follows: >100: extreme, 30–100: very strong, 10–30: strong, 3–10: moderate, 1–3: anecdotal, and 1 or <1: none [20].

## 3. Results and Discussion

### 3.1. Definition of N Nutrient Status

**Figure 1:**
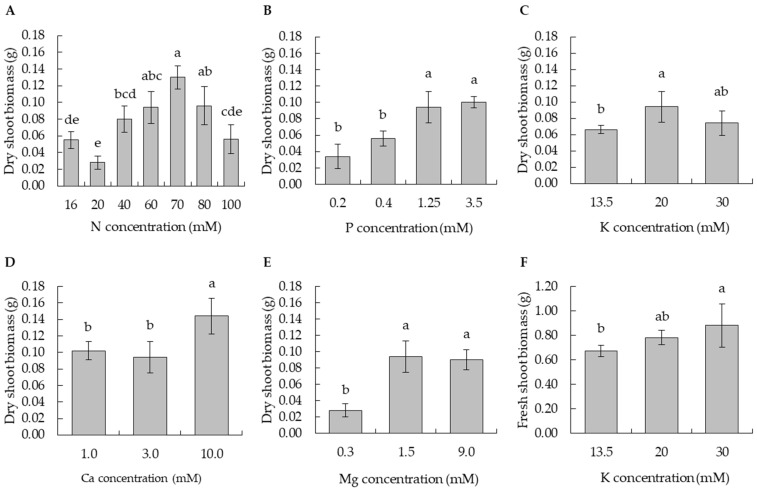
Effect of varying nutrient supplies in MS media on potato plant dry shoot biomass weight: (**A**) nitrogen: N; (**B**) phosphorus: P; (**C**) potassium: K; (**D**) calcium: Ca; (**E**) Magnesium: Mg; and (**F**) K effect on fresh shoot biomass weight. Error bars on bar graphs represent standard deviations. Means separated according to Tukey’s HSD at *p* < 0.05, and bars with same lowercase letters are not statistically different.

**Figure 2:**
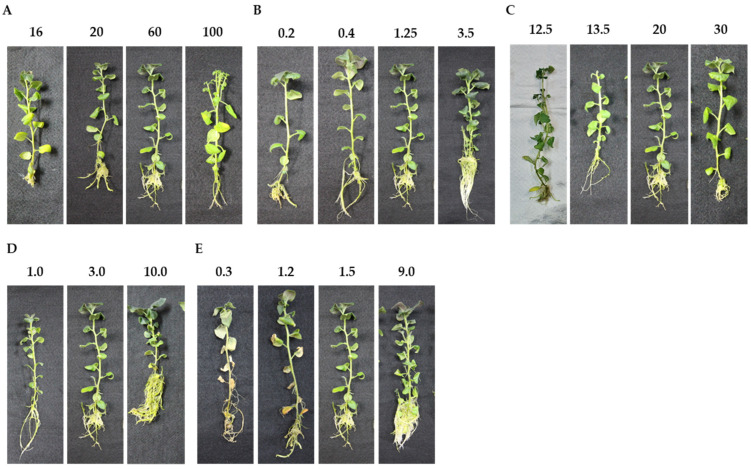
Photographs showing the morphological characteristics of potato plantlets supplied with varying concentrations (mM) of (**A**) nitrogen (N: 16 = deficient, 20–40 = insufficient, 60–80 = optimum, 100 = toxic); (**B**) phosphorus (P: 0.2 = deficient, 0.4 = insufficient, 1.25 = optimum, 3.5 = excessive); (**C**) potassium (K: 12.5 = deficient, 13.5 = insufficient, 20 = optimum, 30 = excessive); (**D**) calcium (Ca: 1.0–3.0 = insufficient, 10.0 = excessive); and (**E**) magnesium (Mg: 0.3–1.2 = deficient, 1.5 = optimum, 9.0 = excessive) < 0.05.

From the dry shoot biomass and morphological symptoms, 16 mM N was defined as deficient, 20 to 40 mM as insufficient, 60 to 80 mM as optimum, and 100 mM as toxic. The maximum shoot dry biomass was observed between 60 and 80 mM, and the dry shoot biomass was significantly reduced in N supplies below 40 mM (Figure 1A). Deficiency symptoms including chlorosis and retarded root growth were observed at 16 mM (Figure 2A). Nguyen et al. [8] reported increased dry shoot biomass with a decreased N supply of 7.5 mM compared to 60 mM, and no chlorotic symptoms were observed in potato cultivar Iwa. Schum et al. [21] reported decreased shoot biomass in 13 out of 17 potato cultivars with an N supply of 7.5 mM compared to 60 mM, and five of the cultivars showed heavy chlorosis. These results affirm that potato N response is cultivar-dependent and a cultivar-specific experiment needs to be run to determine the N levels for a deficient to toxic range.

In contrast, at 100 mM, significantly lower dry shoot biomass was observed, as were symptoms linked to toxicity, including many small leaves, heavy branching, early leaf drying, and minimal lateral root growth (Figure 2A). The N uptake also significantly decreased (Table 2), but shoot N content was significantly higher than that in all other N supply levels (Table 3). This poor growth might be caused by poor root growth. A previous study suggested that N concentrations in solution and in tissue, and thus toxicity level, greatly changed with the N forms and that the optimal range of N was higher with NO_3_^−^ than with NH_4_^+^ nutrition [22]. This suggests that the N toxicity observed in the present study could be due to increased NH_4_^+^ ion concentration in the medium.

When we compared the interaction of N with other elements, we observed a significant decrease in shoot Ca and Mg uptakes in the toxic N range (Table 2), in line with Roosta and Schojoerring’s [23] report of poor growth of cucumber with toxic levels of NH_4_^+^ levels and associated with reduced tissue Ca and Mg contents. Additionally, changes in shoot N uptake had a significant positive interaction with the changes in P, K, Ca, and Mg uptakes (Table 4). Similar findings were reported for N and K in *Actinidia arguta* grown under tissue culture and for N, Ca, and Mg in cucumber grown under hydroponic conditions [23,24]. Therefore, N supply studies in potato plants would require careful consideration of balance with other elements because the reduced uptake of these nutrients can occur before any apparent symptoms.

### 3.2. Definition of P Nutrient Status

Based on the dry shoot biomass and morphological symptoms, 0.2 mM P was defined as deficient, 0.4 mM as insufficient, 1.25 mM as optimum, and 3.5 mM as excessive. At 0.4 mM, a relatively normal growth, stronger stems and leaves, and improved rooting were observed. The relatively normal growth could be due to the slight increases in nutrient uptake (Table 2) caused by an improved root system. However, the dry shoot biomass was significantly reduced at 0.4 mM and was significantly different from 1.25 mM N (Figure 1B). The maximum dry shoot biomass was observed at a concentration of 3.5 mM, which was not significantly different from 1.25 mM. This was the first in vitro study to report the effect of varied P alone and the evaluation of P content in potato plants. Most in vitro studies that have assessed the effect of varied P supply on potato plants were performed in combination with changes in other nutrients [7,25]. In this study, P deficiency symptoms such as weaker stems and darker leaves, as well as fewer and smaller roots, were observed in 0.2 mM N supply (Figure 2B). Similar symptoms were observed by Barben et al. [26] in potato plants grown in P-deficient nutrient solution under hydroponic conditions. The P deficiency was reported to impair both the synthesis and translocation of sugars, which negatively impacts plant growth.

Although the dry shoot biomass did not significantly increase, the P uptake at 3.5 mM significantly increased compared to 1.25 mM, indicating an excessive P uptake (Table 2). This excessive P uptake was more favorable for root growth than for shoot growth (Figure 2B). In a greenhouse study, a similar trend in shoot growth has been reported with a decline in the P use efficiency in shoot growth with high P applications [27]. In addition, the lack of shoot growth could be related to the plant allocating more sugars to the roots, which has been reported in potato plants grown under deficient P [28]. In this study, the same phenomenon also occurred at excessive P supply, as evident from the improved root growth (Figure 2B), suggesting that it occurs not only at deficient but also at excessive P. This correlated with a significant increase in Mg content from 1.25 to 3.5 mM P (Table 3). Mg is known to improve root growth in potato plants [29]. In the field, high P fertilizer applications increase the below-ground biomass [30], reflected in the present study by improved root growth. However, previous studies reported P toxicity in potato cultivar Russet Burbank at an excessive P supply of 1024 µM in a hydroponic solution [26]. This suggests that further increases in P supply above 3.5 mM may produce symptomatic potato plants. In addition, the effect of Mg changes may need careful consideration in P fertilization field studies.

Increases in P supply also increased the uptake of N, K, and Ca in potato shoots (Table 2). The shoot Ca and Mg uptake increased similarly to the P uptake, while for N and K there was no significant difference between 1.25 and 3.5 mM P supply. The changes in shoot P uptake had a significant positive correlation with the changes in N, K, Ca, and Mg uptake (Table 4). Studies on several other crops have reported a positive relationship between P and N [31], and K [32], as well as both positive and negative relationships between P and Ca [13]. Broadley et al. [31] attributed the P and N relationship to their involvement in photosynthesis and protein formation, and that P can be taken up and translocated with K, Ca, and Mg to different parts of the plant.

### 3.3. Definition of K Nutrient Status

Based on the dry shoot biomass and the morphological symptoms, 12.5 mM K was deficient, 13.5 mM was insufficient, 20 mM was optimum, and 30 mM was excessive. The 12.5 mM concentration was not included in further analyses due to poor growth and early defoliation of the plant. The plantlets showed K deficiency symptoms such as lighter green and early yellowing of the lower leaves (Figure 2C). In 13.5 mM, there were significant reductions in dry shoot biomass compared to 20 mM K (Figure 1C). In fact, the insufficient supply of 13.5 mM K was a critical margin below which typical K deficiency symptoms were expressed (Figure 1C and Figure 2C). Similar K deficiency symptoms were observed under K-deficient sand culture. The potato plants exhibited reduced CO_2_ net assimilation and biomass production [33]. This could be attributed to an impaired photosynthesis as well as photoassimilate translocation to newly growing tissues, which negatively impact plant growth [34]. On the other hand, smaller biomass produced at an insufficient K supply of 13.5 mM could also be attributed to the reduction in osmoregulation and cell expansion in the plant [35].

In contrast, at an excess K supply of 30 mM, no increase in dry shoot biomass but a slight increase in fresh shoot biomass was observed (Figure 1C,F), suggesting excessive water uptake in potato plant shoots. This has previously been reported in field-grown potatoes [36] and several other crops [37] where excessive K supply increased water uptake in the plants.

The shoot K uptake showed no significant increase with the increase in MS media K concentrations (Table 2). However, as the media K supply increased, the shoot K content increased significantly (Table 3), which corresponded to an increase in fresh shoot biomass, indicating excessive K content. At excessive K supply, the shoot K uptake did not change while the K content and fresh shoot biomass increased (Table 2 and Table 3, Figure 1C,F). This suggests that the increase in shoot K content creates an osmotic potential due to reduced sugar translocation to the roots resulting in higher water uptake than K uptake. In fact, the lack of sugar translocation to the roots is evident from the poor root growth at excessive K supply (Figure 2C). At both insufficient and excessive K supply, the shoot N uptake decreased (Table 2), indicating that at a lower K supply range, K and N are synergic, while they are antagonistic at an excessive range. Similar findings have been reported in apple dwarf seedlings where N uptake was reduced at both deficient and excessive K supplies [38]. Excessive K supply induces competition between K^+^ ions and NH_4_^+^ ions during the uptake, whereas insufficient K supply hinders the assimilation and translocation of N as observed in *Arabidopsis* [39,40]. Therefore, such interactions are apparent in the present study, and K supply would require careful consideration of N supply. Additionally, a significant positive correlation between changes in K and P uptake in potato shoots was observed (Table 4). This is the first report of such interaction.

### 3.4. Definition of Ca Nutrient Status

From the dry shoot biomass and morphological symptoms, the Ca supplies of 1.0 mM and 3.0 mM were insufficient, and 10.0 mM was optimal. Ozgen et al. [41] observed Ca supply below 3.0 mM showing no effect on plant shoot biomass weight but further decreases below 1.0 mM producing symptomatic potato plants, characterized by increased axillary shoots. This phenomenon was attributed to the loss in apical dominance. In the present study, the reduction in Ca supply from 3.0 to 1.0 mM showed no effect on the shoot biomass, but it reduced root growth (Figure 1D and Figure 2D). The poor root growth could be attributed to the damage in the meristematic regions of the roots due to inadequate Ca supply [42]. A significant increase in the dry shoot biomass accompanied by enhanced plant vigor and root growth was observed at 10.0 mM (Figure 1D and Figure 2D). Upadhyaya et al. [9] reported enhanced plant growth, tuber number, and tuber yield at 9.0 mM Ca under in vitro conditions. On the higher end of Ca concentration, it is still unclear whether Ca can become toxic to potato plants. In fact, we observed that even at 30 mM there was no effect on plant growth when compared to 10 mM, and the plants were asymptomatic, suggesting excessive Ca supply.

While the shoot Ca uptake remained stable between 1.0 and 3.0 mM, it drastically increased at 10 mM Ca supply (Table 2) due to a large increase in shoot Ca content (Table 2) and better shoot growth. The shoot Ca uptake was associated with a steady increase in shoot N, K, and Mg uptake up to 10.0 mM Ca (Table 2), where improved root growth was observed (Figure 2D). Moreover, the Ca uptake was correlated with N, K, and Mg uptake (Table 4). Therefore, future Ca supply studies must consider this synergistic effect with N, K, and Mg supply in order to achieve nutrient balance.

### 3.5. Definition of Mg Nutrient Status

From the dry shoot biomass and morphological symptoms, the supply of 0.3 to 1.2 mM Mg was deficient, 1.5 mM was optimum, and 9.0 mM was excessive. The maximum dry shoot biomass was observed at 1.5 mM, and the dry shoot biomass was significantly reduced at 0.3 mM (Figure 1E). Mg deficiency symptoms such as necrotic lower leaves and fewer and shorter lateral roots were observed in 0.3 and 1.2 mM (Figure 2E). The symptoms of Mg deficiency observed in this study were similar to those reported by Koch et al. [29] in potato plants grown in a Mg-deficient nutrient solution. The authors attributed this to an impaired Mg re-translocation from source to sink tissues, which resulted in the accumulation of sugars in the source organs and sugar starvation in the sink organs. The asymptomatic potato plants produced at 1.5 mM suggested a slight decrease in Mg supply below this level is thus critical. The supply of 9.0 mM decreased the shoot dry biomass slightly when compared to 1.5 mM (Figure 1E), indicating an excess level of Mg, but greatly enhanced lateral root growth when compared to all other Mg levels (Figure 2E). A similar observation has been reported in *Arabidopsis* with an increased Mg supply [43].

The Mg uptake in the shoot increased in accordance with the dry matter up to a point where only the Mg uptake continued to increase, indicating luxury absorption (Table 2 and Table 3). The synergistic increase and significant correlation between N and Mg were observed and appear to be caused by complementary functions within the plant (Table 4). Both Mg and N are involved in chlorophyll synthesis, and N assimilation and uptake require energy from Mg-ATP. Peng et al. [44] observed an increase in the uptake of nitrate but not ammonium in soybean plants grown in high Mg nutrient solution. The shoot P and K uptake were found to be affected only when the Mg supply was deficient (Table 2). This could be due to the poor root growth since both P and K are taken up by diffusion which requires well-developed roots [45,46]. These nutrient changes suggest that the Mg supply must take into consideration N, P, and K supplies in order to achieve balanced plant nutrition.

## 4. Conclusions

The N, P, K, Ca, and Mg uptake in the potato plants was consistent with the growth and morphological symptoms reported in the literature. Very few studies have considered correlations in element content in potato plants; our findings show that drastic interactions among the nutrient elements exist. Therefore, there is a need for careful consideration of these interactions when growing potato plants for experiments as well as in fertilizer management under field conditions. This approach offers a potential platform for the acquisition of precise data and conducting further research on biotic and abiotic stresses in potatoes, which can be adapted in field research. However, depending on the potato cultivar and nutrient interactions, additional adjustments may be required to maximize the effectiveness of this method. As part of integrated vector control management in potatoes, we plan to subject these potato plants with insufficient and excessive nutrition status to biotic stresses such as aphid feeding behaviors.

## Figures and Tables

**Table 1:** Amount of salts that were varied in MS media for propagation of potato plantlets with insufficient and high N, P, K, Ca, and Mg nutrient status for plant nutrient effect bioassays.

	Concentration	NH_4_NO_3_	KNO_3_	KH_2_PO_4_	KCl	Ca(NO_3_)_2_ 4H_2_O	CaCl_2_ 2H_2_O	MgCl_2_ 6H_2_O	MgSO_4_ 7H_2_O	KI
	(mM)	(g L^−1^)								
Standard ^a^		1.65	1.90	0.17			0.440		0.37	0.00083
N	20	0.775		0.17	1.39		0.440		0.37	0.00083
	70	1.925	1.90	0.17			0.440		0.37	0.00083
P	0.4	1.65	1.90	0.058	0.062		0.440		0.37	0.00083
	3.5	1.74	1.67	0.479			0.440		0.37	0.00083
K	13.5	1.65		0.17	0.921	0.0312	0.421		0.37	0.00083
	30	1.65	1.90	0.17	0.752		0.440		0.37	0.00083
Ca	1.0	1.65	1.90	0.17			0.147		0.37	0.00083
	10.0	1.65	1.90	0.17			1.467		0.37	0.00083
Mg	1.5	1.65	1.90	0.17			0.440		0.37	0.00083
	9.0	1.65	1.90	0.17			0.440	1.52	0.37	0.00083

^a^ as in standard MS medium, NH_4_NO_3_: ammonium nitrate, KNO_3_: potassium nitrate, KH_2_PO_4_: potassium dihydrogen phosphate, KCl: potassium chloride, Ca(NO_3_)_2_ 4H_2_O: calcium nitrate tetrahydrate, CaCl_2_ 2H_2_O: calcium chloride dihydrate, MgCl_2_ 6H_2_O: magnesium chloride hexahydrate.

**Table 2:** Effect of changes in nutrient element concentrations in MS medium on nutrient uptake in shoots of potato plants in vitro.

	Medium Element Concentration	N			P			K			Ca			Mg		
Element	(mM)	(mg Plantlet^−^^1^)														
N	20	1.90	±	0.40 c	0.26	±	0.06 b	0.73	±	0.17 c	0.26	±	0.04 c	0.10	±	0.02 b
	40	4.36	±	0.71 b	0.51	±	0.13 a	3.61	±	0.39 a	0.44	±	0.06 b	0.19	±	0.03 a
	60 ^a^	6.03	±	1.15 ab	0.59	±	0.13 a	3.96	±	1.40 a	0.43	±	0.07 b	0.19	±	0.06 a
	70	7.39	±	0.62 a	0.45	±	0.03 ab	3.02	±	0.28 ab	0.58	±	0.07 a	0.20	±	0.02 a
	80	7.50	±	1.89 a	0.66	±	0.12 a	2.53	±	1.08 ab	0.30	±	0.07 c	0.15	±	0.05 ab
	100	4.98	±	1.43 b	0.44	±	0.15 ab	1.77	±	0.38 bc	0.21	±	0.06 c	0.10	±	0.03 b
P	0.2	2.22	±	0.85 c	0.14	±	0.04 c	1.14	±	0.52 b	0.14	±	0.07 c	0.06	±	0.02 c
	0.4	3.75	±	0.44 b	0.24	±	0.05 c	1.83	±	0.29 b	0.24	±	0.04 c	0.10	±	0.01 c
	1.25 ^a^	6.03	±	1.15 a	0.59	±	0.13 b	3.96	±	1.40 a	0.43	±	0.07 b	0.19	±	0.06 b
	3.5	7.36	±	0.59 a	1.35	±	0.17 a	4.71	±	0.42 a	0.62	±	0.05 a	0.25	±	0.04 a
K	13.5	4.18	±	0.46 b	0.48	±	0.14 a	2.53	±	0.73 a	0.34	±	0.07 a	0.15	±	0.02 a
	20.0 ^a^	6.03	±	1.15 a	0.59	±	0.13 a	3.96	±	1.40 a	0.43	±	0.07 a	0.19	±	0.06 a
	30.0	4.33	±	0.89 b	0.57	±	0.08 a	3.94	±	0.91 a	0.32	±	0.08 a	0.15	±	0.03 a
Ca	1.0	6.09	±	0.57 b	0.52	±	0.03 a	2.07	±	0.42 b	0.20	±	0.02 b	0.15	±	0.01 b
	3.0 ^a^	6.03	±	1.15 b	0.59	±	0.13 a	3.96	±	1.40 a	0.43	±	0.07 b	0.19	±	0.06 ab
	10.0	8.61	±	1.17 a	0.63	±	0.07 a	4.57	±	0.49 a	1.56	±	0.29 a	0.25	±	0.04 a
Mg	0.3	1.60	±	0.30 c	0.33	±	0.14 b	0.40	±	0.15 b	0.28	±	0.09 b	0.06	±	0.02 c
	1.5 ^a^	6.03	±	1.15 b	0.59	±	0.13 a	3.96	±	1.40 a	0.43	±	0.07 a	0.19	±	0.06 b
	9.0	8.04	±	1.22 a	0.59	±	0.07 a	3.50	±	0.14 a	0.33	±	0.04 ab	0.81	±	0.11 a

^a^ Standard as in MS medium [6]. n = 5. Means are presented with ± standard deviations. Means separated according to Tukey’s HSD at *p* < 0.05.

**Table 3:** Effect of changes in nutrient element concentrations in MS medium on nutrient content in shoots of potato plants in vitro.

	Medium Element Concentration	N			P			K			Ca			Mg		
Element	(mM)	(g kg^−^^1^)														
N	20	64.4	±	3.4 c	9.8	±	2.6 a	27.7	±	8.2 b	9.7	±	1.9 a	3.7	±	0.8 a
	40	55.0	±	5.3 d	6.6	±	1.7 b	46.0	±	5.9 a	5.5	±	0.8 b	2.3	±	0.2 b
	60 ^a^	64.4	±	3.4 c	7.0	±	0.9 ab	45.6	±	4.8 a	4.3	±	0.5 bc	2.1	±	0.1 bc
	70	57.0	±	3.5 cd	3.5	±	0.4 c	23.4	±	3.2 b	4.4	±	0.6 bc	1.5	±	0.2 c
	80	78.0	±	4.1 b	6.9	±	0.9 b	25.6	±	4.4 b	3.1	±	0.3 c	1.5	±	0.1 c
	100	89.2	±	3.4 a	8.0	±	1.3 ab	32.5	±	5.3 b	3.6	±	0.1 c	1.7	±	0.1 bc
P	0.2	66.6	±	5.8 ab	4.3	±	1.4 c	33.3	±	2.8 b	4.0	±	0.3 b	1.8	±	0.2 bc
	0.4	67.4	±	4.7 ab	4.4	±	1.2 c	32.7	±	2.8 b	4.2	±	0.3 b	1.7	±	0.2 c
	1.25 ^a^	64.4	±	3.4 b	7.0	±	0.9 b	45.6	±	4.8 a	4.3	±	0.5 b	2.1	±	0.1 b
	3.5	73.6	±	2.5 a	13.4	±	1.2 a	47.4	±	6.3 a	6.3	±	0.5 a	2.5	±	0.3 a
K	13.5	63.2	±	3.0 ab	7.3	±	1.7 a	38.0	±	8.9 b	5.2	±	0.8 a	2.3	±	0.2 a
	20.0 ^a^	64.4	±	3.4 a	7.0	±	0.9 a	45.6	±	4.8 ab	4.3	±	0.5 a	2.1	±	0.1 a
	30.0	58.6	±	2.6 b	7.9	±	1.2 a	53.7	±	8.6 a	4.4	±	0.3 a	2.1	±	0.1 a
Ca	1.0	59.8	±	2.6 a	5.2	±	0.5 b	20.2	±	3.2 c	1.9	±	0.1 c	1.5	±	0.1 c
	3.0 ^a^	64.4	±	3.4 a	7.0	±	0.9 a	45.6	±	4.8 a	4.3	±	0.5 b	2.1	±	0.1 a
	10.0	60.0	±	2.5 a	4.4	±	0.6 b	32.2	±	5.1 b	10.8	±	1.0 a	1.7	±	0.2 b
Mg	0.3	47.2	±	6.4 b	11.7	±	2.0 a	14.2	±	3.2 b	9.8	±	0.7 a	2.1	±	0.2 b
	1.5 ^a^	64.4	±	3.4 a	7.0	±	0.9 b	45.6	±	4.8 a	4.3	±	0.5 b	2.1	±	0.1 b
	9.0	66.6	±	6.1 a	6.6	±	0.6 b	39.4	±	4.4 a	3.6	±	0.3 b	9.0	±	0.7 a

^a^ Standard as in MS medium [6]. n = 5. Means are presented with ± standard deviations. Means separated according to Tukey’s HSD at *p* < 0.05.

**Table 4:** Bayesian Pearson’s correlations among N, P, K, Ca, and Mg uptake in shoot of potato plants as affected by individual nutrient concentrations in MS medium in vitro.

Change in Shoot Nutrient Uptake	Shoot Nutrient Uptake	n	Pearson’s r	*p* Value	BF_10_	Lower 95% CI	Upper 95% CI
N	P	30	0.723	<0.001 ^†††^	7231.98 ***	0.461	0.850
	K	30	0.554	0.001 ^††^	56.03 **	0.225	0.747
	Ca	30	0.520	0.003 ^††^	28.12 *	0.183	0.724
	Mg	30	0.541	0.002 ^††^	42.89 **	0.209	0.738
P	N	20	0.870	<0.001 ^†††^	54,778.27 ***	0.647	0.944
	K	20	0.825	<0.001 ^†††^	5984.41 ***	0.552	0.922
	Ca	20	0.935	<0.001 ^†††^	11,040,000.00 ***	0.805	0.974
	Mg	20	0.908	<0.001 ^†††^	727,016.73 ***	0.735	0.961
K	N	15	0.382	0.160	1.43	0.031	0.713
	P	15	0.856	<0.001 ^†††^	1152.63 ***	0.537	0.947
	Ca	15	0.384	0.157	1.45	0.032	0.715
	Mg	15	0.363	0.183	1.29	0.029	0.704
Ca	N	15	0.881	<0.001 ^†††^	3138.88 ***	0.599	0.957
	P	15	0.399	0.070	1.58	0.034	0.722
	K	15	0.629	0.006 ^††^	11.24 *	0.152	0.839
	Mg	15	0.743	<0.001 ^†††^	62.26 **	0.310	0.895
Mg	N	15	0.812	<0.001 ^†††^	291.83 ***	0.440	0.927
	P	15	0.533	0.020 ^†^	4.23	0.079	0.791
	K	15	0.523	0.023 ^†^	3.87	0.073	0.786
	Ca	15	0.060	0.415	0.38	0.009	0.554

^†^ α = 5 %, ^††^ α = 1 %, ^†††^ α = 0.1 %. * BF_10_ > 10, ** BF_10_ > 30, *** BF_10_ > 100, CI: Confidence interval.

## Data Availability

Not applicable.
